# Modelling the cost-effectiveness of pay-for-performance in primary care in the UK

**DOI:** 10.1186/s12916-018-1126-3

**Published:** 2018-08-29

**Authors:** Ankur Pandya, Tim Doran, Jinyi Zhu, Simon Walker, Emily Arntson, Andrew M. Ryan

**Affiliations:** 1 Department of Health Policy and Management, Harvard T.H. Chan School of Public Health, 718 Huntington Ave, 2nd Floor, Boston, MA 02115 USA; 2 Department of Health Sciences, University of York, Heslington, York, UK; 3 Center for Health Decision Science, Harvard T.H. Chan School of Public Health, Boston, MA USA; 4 Centre for Health Economics, University of York, Heslington, York, UK; 5 Department of Health Management and Policy, University of Michigan School of Public Health, Ann Arbor, MI USA

## Abstract

**Background:**

Introduced in 2004, the United Kingdom’s (UK) Quality and Outcomes Framework (QOF) is the world’s largest primary-care pay-for-performance programme. Given some evidence of the benefits and the substantial costs associated with the QOF, it remains unclear whether the programme is cost-effective. Therefore, we assessed the cost-effectiveness of continuing versus stopping the QOF.

**Methods:**

We developed a lifetime simulation model to estimate quality-adjusted life years (QALYs) and costs for a UK population cohort aged 40–74 years (*n* = 27,070,862) exposed to the QOF and for a counterfactual scenario without exposure. Based on a previous retrospective cross-country analysis using data from 1994 to 2010, we assumed the benefits of the QOF to be a change in age-adjusted mortality of −3.68 per 100,000 population (95% confidence interval –8.16 to 0.80). We used cost-effectiveness thresholds of £30,000/QALY, £20,000/QALY and £13,000/QALY to determine the optimal strategy in base-case and sensitivity analyses.

**Results:**

In the base-case analysis, continuing the QOF increased population-level QALYs and health-care costs yielding an incremental cost-effectiveness ratio (ICER) of £49,362/QALY. The ICER remained >£30,000/QALY in scenarios with and without non-fatal outcomes or increased drug costs, and under differing assumptions about the duration of QOF benefit following its hypothetical discontinuation. The ICER for continuing the programme fell below £30,000/QALY when QOF incentive payments were 36% lower (while preserving QOF mortality benefits), and in scenarios where the QOF resulted in substantial reductions in health-care spending or non-fatal cardiovascular disease events. Continuing the QOF was cost-effective in 18%, 3% and 0% of probabilistic sensitivity analysis iterations using thresholds of £30,000/QALY, £20,000/QALY and £13,000/QALY, respectively.

**Conclusions:**

Compared to stopping the QOF and returning all associated incentive payments to the National Health Service, continuing the QOF is not cost-effective. To improve population health efficiently, the UK should redesign the QOF or pursue alternative interventions.

**Electronic supplementary material:**

The online version of this article (10.1186/s12916-018-1126-3) contains supplementary material, which is available to authorized users.

## Background

Introduced in 2004, the United Kingdom’s Quality and Outcomes Framework (QOF) is the world’s largest primary-care pay-for-performance programme. The QOF links up to 25% of general practitioners’ income to performance on a wide range of quality indicators related to clinical management of common chronic conditions, organisation of care and patient experience [1]. This supplements existing payments to practices, which are largely provided through capitation payments. Research on the QOF suggests that the programme accelerated improvement for the incentivised indicators in the 3 years following its implementation [2]. However, this improvement appeared to attenuate over time [3–5]. A recent analysis also found that the QOF did not significantly improve mortality for disease areas targeted under the programme [6].

The QOF is subject to annual review, with changes agreed in negotiations between National Health Service (NHS) Employers and the British Medical Association’s General Practitioners Committee, informed by indicator development work conducted by the National Institute for Health and Care Excellence. In 2014/15, 40 indicators—accounting for 35% of the value of total incentive payments—were removed from the scheme without replacement, with most of the associated resource used to increase capitation payments [7]. In 2016/17, QOF was discontinued altogether in Scotland and funding was transferred to capitation payments [8]. QOF continues in England, Wales and Northern Ireland, although options for reform or replacement are being considered.

Despite the large costs of the QOF and other pay-for-performance programmes, almost no evidence exists on their cost-effectiveness and how this compares to other system-level interventions to improve longevity [5, 9, 10]. Pay-for-performance programmes introduce additional economic costs to the health-care system, which could have been spent on other health interventions or policies. A cost-effectiveness analysis, the standard method for assessing value for money, can be used to determine whether additional spending on pay-for-performance is worth the health gains produced by these policies. Although a cost-effectiveness analysis has previously been conducted for other pay-for-performance programmes in the UK, decisions on the development or discontinuation of QOF have not been informed by reliable estimates of cost-effectiveness [11]. A systematic review of pay-for-performance programmes found that, despite the promise of cost-effective financial incentives, convincing evidence of the cost-effectiveness of pay-for-performance was lacking [12]. Previous attempts to estimate the cost-effectiveness of pay-for-performance have extrapolated evidence from randomised trials, rather than using direct evidence of its effectiveness on outcomes [4, 9, 13]. These approaches are limited because results from randomised trials may not generalise to the older, sicker patients who are typically excluded from trials [6].

In this study, we address this knowledge gap by evaluating the cost-effectiveness of the QOF under various assumptions around the programme’s benefits and costs.

## Methods

### Overview

**Fig. 1:**
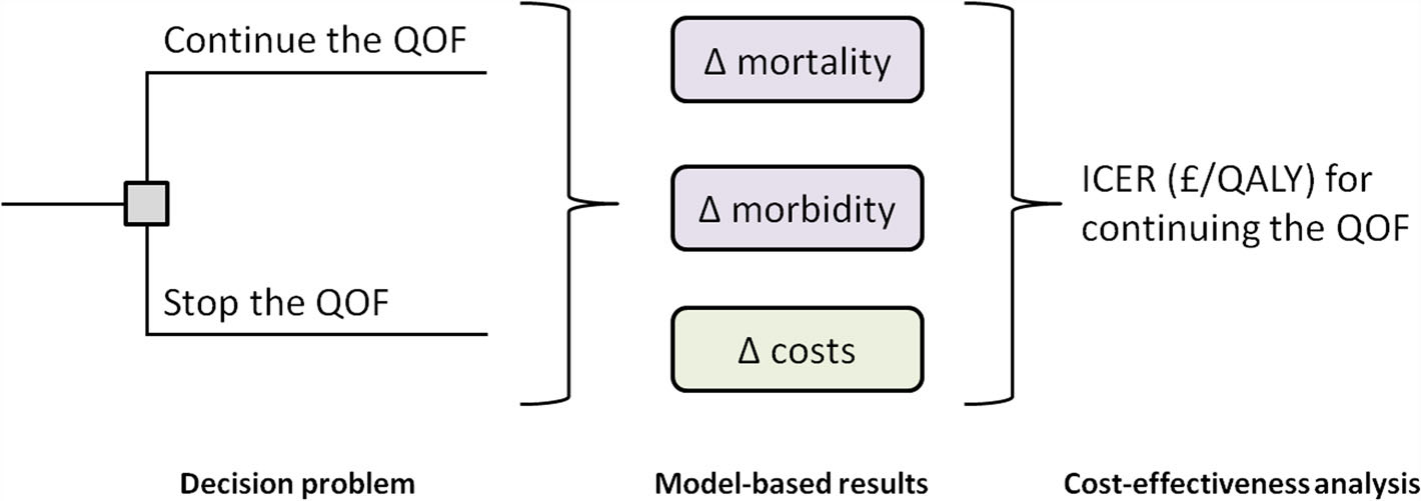
Conceptual diagram of the cost-effectiveness analysis. Individuals enter the simulation model and are assigned to one of two QOF scenarios. The model estimates the impact of continuing or stopping the QOF on mortality, morbidity and QOF-related cost outcomes. The trade-offs between quality-adjusted life years (QALYs) and costs are evaluated by calculating an incremental cost-effectiveness ratio (ICER) for continuing the QOF. ICER incremental cost-effectiveness ratio, QALY quality-adjusted life year, QOF Quality and Outcomes Framework

We developed a computer-based simulation model to estimate the QOF-related lifetime health effects and costs for a representative UK general population cohort aged 40–74 years (*n* = 27,070,862) exposed to two competing policy alternatives: (1) continuing the QOF and (2) stopping the QOF. We compared the trade-off between lifetime discounted health effects, quantified using quality-adjusted life years (QALYs), and QOF-related health-care costs using an incremental cost-effectiveness analysis from a NHS (health-care payer) perspective. The simulation model discounts health effects and costs using a rate of 3.5% [14]. An overview of our methodology is shown in Fig. 1.

### Simulation model and population

The overall goal for our model-based analysis was to estimate the impact of continuing the QOF (compared to stopping the QOF) on lifetime QALYs and costs. The decision of whether or not to continue the QOF will have the most impact on the specific diseases targeted by the QOF. For instance, the lifetime QALYs gained would be greater if the QOF mortality reductions were concentrated in healthy children as opposed to older persons with multiple chronic conditions. Of all the conditions actually included in the QOF, cardiovascular diseases and associated risk conditions (coronary heart disease, stroke, diabetes and hypertension) collectively attract the largest incentives and account for the majority of non-cancer deaths (although cancer is included in the QOF, it only receives weak incentives) [6, 15–17]. In 2005, payments of up to £15,125 were available for the average family practice across 15 ischaemic heart disease and heart failure indicators, but only £1500 was available across two cancer indicators [6]. Therefore, we assumed that the QOF benefits were concentrated in individuals with cardiovascular disease (aged 40–74 years at baseline) in our base-case analysis, but relaxed this assumption in sensitivity analyses (including a scenario where the QOF equally benefited all individuals aged 0–74 years with and without disease). We needed to make these assumptions around which population segments would benefit from the QOF because our main effectiveness estimate for the QOF did not specify in whom the mortality benefits were concentrated, but rather reported an overall age-adjusted mortality reduction of 3.68 per 100,000 (across those aged 0–74 years) [6].

We created a hypothetical cohort that would be affected by the QOF using age group- and sex-specific population sizes for the UK, further dividing this population into those with and those without prevalent cardiovascular disease. We used Office for National Statistics data for sex-specific population sizes in 5-year age categories (e.g., 2,255,562 females aged 50–54 years at baseline) and sex- and age-specific cardiovascular disease prevalence estimates from British Heart Foundation data (e.g. 8.4% of females aged 50–54 years at baseline had prevalent cardiovascular disease, ICD-10 codes I00–I99) [15, 16]. Our model uses annual cycles to project the life expectancy, quality-adjusted life expectancy and QOF-related costs for a representative UK cohort until death. Each model population segment faces a risk of death in each model cycle based on the sex, age and cardiovascular disease status of the group being simulated, with additional mortality risk adjustments to account for the absence of the QOF for the non-QOF strategy (i.e. the current life tables include the effects of the QOF, so we increased the risk of death in the life tables when simulating the non-QOF strategy). Those with cardiovascular disease faced higher mortality risks and reduced quality of life compared to those without cardiovascular disease. To estimate mortality risks for those with cardiovascular disease, we multiplied the annual mortality risk all-cause life tables (i.e. the age-specific annual risks of death from any cause) by 1.6 (for men) or 2.1 (for women). These age-adjusted mortality ratios were based on an analysis of linked hospital discharge and 7-year mortality follow-up data from survivors of first acute myocardial infarction in England from 2004 to 2010 [18]. We subtracted cardiovascular disease mortality rates from all-cause life tables to estimate mortality risks for those without cardiovascular disease [18]. The model sums all health effects and costs accrued for each population segment, yielding population-level results. Table 1 shows all model inputs and data sources. The model is programmed in Microsoft Excel with Visual Basic for Applications and is included as Additional file 1.

**Table 1:** Model variables with base-case values and ranges used in sensitivity analyses

Variable	Base-case value	Sensitivity analysis range	Source(s)
QOF mortality benefit (age- and sex-adjusted per 100,000)	−3.68	−8.16 to 0.80	[6]
Adjusted QOF mortality benefit (for those with age > 40 years with CVD)	−58.93	−130.57 to 12.81	Calculated
All-cause age- and sex-specific mortality (and age and sex demographics)	Life table	Not applicable	[45]
CVD prevalence, males (aged 45–64 years, aged 65–74 years)	14.6%, 28.5%	+/− 20%	[16]
CVD prevalence, females (aged 45–64 years, aged 65–74 years)	8.4%, 22.5%	+/− 20%	[16]
CVD mortality multiplier (male, female)	1.6, 2.1	+/−20%	[18]
CVD utility	0.796	+/− 20%	[21]
Non-fatal-to-fatal CVD events averted (ratio)	1.63	0–10	[20]
QOF annual population-level incentive costs	£1,396,843,151	£0–2,000,000,000	Country-specific sources [24, 26, 27]
QOF effect on utilisation costs per £ spent on incentives	£0.011	-£1-£1	[29, 30]
Acute CVD event costs (i.e. costs within first year of CVD event)	£10,871	+/−20%	[31]
Chronic CVD event costs (i.e. annual costs for all years after first year)	£3282	+/− 20%	[31]
Average NHS costs by age	Age-based table	£0 to + 100%	[32]
Discount rate	3.5%	0–5%	[14]

*QOF* Quality and Outcomes Framework, *CVD* cardiovascular disease, *NHS* National Health Service

### QOF mortality effects

The mortality increase from stopping the QOF was estimated from a previous difference-in-difference analysis [6]. In their study, Ryan et al. created a synthetic control group as a weighted combination of countries previously characterised as having a high-income epidemiological profile. Based on their study, the QOF programme was associated with a change in age- and sex-adjusted mortality from the major contributors to population mortality targeted by the QOF (ischaemic heart disease, hypertension, stroke, diabetes, chronic kidney disease, asthma and chronic pulmonary disease) of −3.68 per 100,000 population (95% confidence interval of −8.16 to 0.80). This estimate was based on age-adjusted mortality rates for populations aged 0–74 years. Since QOF mortality benefits would be concentrated in older individuals with prevalent disease, we adjusted the mortality effect used in our model by back-calculating mortality change (58.9 per 100,000 population) in older individuals (aged 40–74 years, *n* = 27,070,862) with prevalent cardiovascular disease that would result in the Ryan et al. general population result. In other words, a 58.9 per 100,000 mortality decrease concentrated exclusively in individuals aged 40–74 years with cardiovascular disease would result in a 3.68 per 100,000 mortality effect in all individuals (aged 0–74 years among individuals with and without cardiovascular disease, *n* = 59,385,341). We performed a sensitivity analysis using a 3.68 per 100,000 mortality decrease for all individuals (aged 0–74 years) to assess the impact of these two contrasting assumptions (mortality effect concentrated in those aged 40–74 years versus mortality effect spread out among those aged 0–74 years) on the model results. While either assumption had QOF mortality effects stopping at age 74 years (due to our data source [6]), the model cohort was modelled until death in all analyses.

In the base-case analyses, we assumed the QOF-related mortality benefit was immediately lost if the QOF were discontinued. In the sensitivity analyses, we modelled different durations over which the mortality benefit of the QOF waned if the programme were discontinued. In these scenarios, we assumed linear declines in the QOF mortality benefit from first year in the model to a time in the future (1, 3, 5 or 10 years from the model start), at which point the mortality benefit from the QOF would equal zero.

### QOF non-fatal effects (i.e. QOF morbidity effects)

Best practice in a cost-effectiveness analysis is to include all health outcomes (fatal and non-fatal) that differ from the choice of a particular strategy over another [19]. While it is likely that the QOF resulted in changes in both fatal and non-fatal outcomes, the Ryan et al. study did not include non-fatal outcomes due to data restrictions. Given the QOF focus on managing cardiovascular disease risk, we applied a ratio of non-fatal cardiovascular disease events averted for every fatal event averted from the QOF based on prevented cardiovascular disease events from a meta-analysis of statin trial data from 90,056 participants [20]. First, we calculated the differences in non-fatal coronary heart disease events between statin and placebo arms in the meta-analysis (1789 – 2460) and divided that difference by the difference in coronary heart disease deaths (1548 – 1960), which resulted in a ratio of non-fatal-to-fatal coronary heart disease events averted of 1.63. We multiplied this ratio by the difference in mortality between the QOF and non-QOF counterfactuals in the model to estimate the number of non-fatal cardiovascular disease events averted by the QOF in each model cycle. The model incorporates morbidity in the QALY calculation by applying utility values (see *Health-related quality-of-life* section below) to the non-fatal cardiovascular disease events averted by the QOF. We performed sensitivity analyses excluding non-fatal health effects attributable to the QOF in the model in addition to varying the value of the non-fatal-to-fatal ratio used in the model.

### Health-related quality of life

The main effectiveness measure for our analysis was lifetime cohort-level QALYs. The model calculates QALYs by multiplying the number of individuals alive in a given year by a utility value, which quantifies morbidity. The model assigns utility values ranging between 0 (death) and 1 (perfect health) for each year based on age and cardiovascular disease status. Older individuals and those with cardiovascular disease have lower utility values than younger individuals and those without cardiovascular disease, respectively. Utility values were estimated from the results of the EuroQOL 5 Dimensions (EQ-5D) questionnaire from the Medical Expenditure Panel Survey (US-based descriptive responses to the EQ-5D were combined with UK community-based preference weights to calculate utility values for the UK) [21–23]. For a given age (40–100 years), population segments with cardiovascular disease were assigned the minimum utility for cardiovascular disease (0.796) and age-based utilities (ranging from 0.691–0.848). Population segments without cardiovascular disease were assigned the age-based utility values [23].

### QOF costs

We included two major QOF-related cost categories: (1) incentive costs and (2) costs related to changes in health-care utilisation as a result of QOF incentives. We estimated total incentive costs over the first 7 years of the programme at £1,396,843,151 per year based on remuneration data from the four constituent countries of the UK (see Table 4 in the Appendix) [24–27]. These costs include payments for all domains (clinical, organisational and patient experience) and are considered incremental (i.e. new) costs to the health system, as opposed to reallocating existing funds within the health system, as they represent additional resources the UK government committed to primary care in 2004 [28].

Given the focus of the QOF incentives on disease management in the cardiovascular domain, we assumed changes in health-care utilisation driven by the QOF would be concentrated in the utilisation of cardiovascular disease drugs (targeting cholesterol, blood pressure and diabetes). We estimated changes in drug utilisation based on an observational study in Scotland, which reported an average increase in defined daily doses (DDDs) per prescribing unit (PU) per month of QOF-related drugs of 3.79. Among these drugs, cardiovascular disease drugs accounted for 3.47 of the average increase (DDD per PU, 89% of the total increase across all drugs mentioned or implied by the QOF) [29]. For these costs, we used separated DDD per PU per month estimates from that analysis for five cardiovascular disease drug classes (lipid-regulating, renin-angiotensin system, thiazides and related diuretics, oral antidiabetics and antiplatelets) in conjunction with weighted averages of drug class prices (Table 5 in the Appendix) to estimate an overall annual increase in QOF-related drug costs of £15,692,470 (i.e. a ratio of 0.011 of increased drug utilisation per £1 spent on incentives) [30]. We allowed this value to be negative (i.e. a cost saving) in a sensitivity analysis to evaluate the possibility that QOF incentives might result in a net decrease in overall health-care spending from averted disease events and follow-up spending, by varying the ratio from −1 to 1 in a one-way sensitivity analysis. In the model, averted cardiovascular disease events (fatal and non-fatal) resulted in cost savings from averted health-care utilisation. We estimated the costs of averted cardiovascular disease events in the first year of the event (£10,871) and all subsequent years (£3282) from a previous analysis of linked UK cohort datasets with utilisation and cost data [31]. We modelled future average age-based annual health-care costs based on English NHS data and performed separate sensitivity analyses where these costs were excluded and doubled [32]. All costs in our analyses are reported in 2016 pounds sterling (£).

### Base-case cost-effectiveness analysis

We used conventional incremental cost-effectiveness analysis methods to calculate an incremental cost-effectiveness ratio (ICER) for continuing the QOF programme compared to stopping the QOF. We used cost-effectiveness thresholds of £30,000/QALY, £20,000/QALY and £13,000/QALY. These thresholds are based on current National Institute for Health and Care Excellence recommendations (£20,000–30,000/QALY) and a 2015 Claxton et al. study that estimated an empirically based opportunity cost for health-care spending (i.e. a cost-effectiveness threshold that estimates the amount of money, at the margin of health-care spending, it would take to produce one additional QALY) in the UK (£13,000/QALY) [33]. We also calculated the opportunity costs of continuing the QOF in terms of population-level incremental net health benefit, which converts incremental costs into QALYs using a cost-effectiveness threshold and subtracts these opportunity costs from QALYs gained as a result of continuing the QOF. In other words, the incremental net health benefit captures the health gains to individuals who benefit from funding being given to an intervention minus the population health forgone as a result of committed resources being unavailable for other individuals’ health care. The health forgone by other individuals was estimated based on the cost-effectiveness thresholds noted above [specifically, the QALYs forgone (incremental net health benefit) equal the incremental costs divided by the cost-effectiveness threshold] [34].

### Sensitivity analyses

We performed four types of sensitivity analyses: (1) scenario analyses for the inclusion of non-fatal health effects and increased drug utilisation as a result of the QOF and the duration of the QOF mortality benefit if the QOF were discontinued, (2) one-way sensitivity analyses with high and low values for all model inputs, (3) a two-way sensitivity analysis for different combinations of the levels of QOF incentive payments and the QOF mortality benefit and (4) a probabilistic sensitivity analysis, where we assessed the statistical uncertainty in the QOF mortality benefit (which was based on an estimate that had a 95% confidence interval of −0.80 to 8.16) by drawing 1000 random values for the QOF mortality benefit from a normal probability distribution [35]. The probabilistic sensitivity analysis results for each of the 1000 iterations were used to construct a cost-effectiveness acceptability curve showing the probability that continuing the QOF was cost-effective while varying the cost-effectiveness threshold from £0–200,000/QALY.

## Results

### Base-case results

Continuing the QOF programme indefinitely would result in more life years, QALYs and QOF-related costs compared to stopping the QOF (Table 2). Compared to stopping the QOF, continuing the QOF had an ICER of £49,362/QALY under base-case assumptions (non-fatal outcomes and increased drug costs included, with instant changes in the QOF mortality benefit if the QOF is discontinued, Table 2). The population opportunity cost of continuing the QOF (i.e. the incremental net health benefit) ranged from 226,109 to 979,917 QALYs lost, assuming cost-effectiveness thresholds of £30,000/QALY to £13,000/QALY, respectively (Table 6 in the Appendix).

**Table 2:** Base-case population-level results and incremental cost-effectiveness analysis of continuing the QOF vs. stopping the QOF

	Undiscounted			Discounted			
	life years	QALYs	QOF-related costs	life years	QALYs	QOF-related costs	ICER (£/QALY)
Stopping the QOF	78,805,931	59,562,301	£0	52,426,797	39,966,375	£0	Reference
Continuing the QOF	79,433,681	60,237,416	£25,881,916,480	52,750,331	40,316,707	£17,293,239,670	49,362
Delta	627,750	675,114	£25,881,916,480	323,534	350,332	£17,293,239,670	–

*QOF* Quality and Outcomes Framework, *QALY* quality-adjusted life year, *ICER* incremental cost-effectiveness ratio

### Scenario analyses

Table 3 shows the matrix of ICERs for the QOF for every combination of assumptions related to the inclusion of non-fatal outcomes, the inclusion of increased drug costs and waning of the QOF benefit. The ICER remained greater than £30,000/QALY for every combination of assumptions. The ICER for continuing the QOF was £42,296/QALY when we assumed all individuals aged 40–74 years experienced the health benefits of the QOF (as opposed to only those with prevalent cardiovascular disease), and £31,089/QALY when the benefits of the QOF were spread out to all individuals (with and without disease) aged 0–74 years (these findings can be reproduced using the simulation model attached as Additional file 1).

**Table 3:** Cost-effectiveness ratios (£/QALY) for continuing the QOF versus stopping the QOF under various model scenarios

QOF effects beyond mortality		How long QOF mortality benefit is sustained if QOF discontinued**				
Non-fatal outcomes	Increased drug costs	No waning	1-year waning	3-year waning	5-year waning	10-year waning
Included	Included	49,362*	51,970	57,616	63,765	81,428
Included	Not included	48,768	51,347	56,931	63,011	80,478
Not included	Included	80,515	84,323	92,565	101,535	127,281
Not included	Not included	79,657	83,424	91,575	100,446	125,907

*QOF* Quality and Outcomes Framework, *QALY* quality-adjusted life year

*Base-case scenario: non-fatal outcomes and increased drug costs included and instant changes in the QOF mortality benefit if the QOF is discontinued

**In waning scenarios, we assumed linear declines in the QOF mortality benefit from the first year in the model to a time in the future (1, 3, 5 or 10 years from the model start), at which point the mortality benefit from the QOF would equal zero

### One-way sensitivity analyses

The ICER was only below alternative National Institute for Health and Care Excellence thresholds of £30,000/QALY, £20,000/QALY and £13,000/QALY when, for every £1 spend on QOF incentives, £0.36, £0.55 or £0.68 were saved as a result of net cost reductions (for example, from averted cardiovascular disease events; Figure 4 in the Appendix). Similarly, the ICER was below the thresholds of £30,000/QALY, £20,000/QALY and £13,000/QALY when the ratios for non-fatal-to-fatal cardiovascular disease events averted were 3.7 (225% of the base-case value), 5.5 (335% of the base-case value) and 7.3 (450% of the base-case value, Figure 5 in the Appendix).

The cost-effectiveness results were robust (ICERs within £40,000–65,000/QALY) in the following sensitivity analyses (with high and low parameter ranges as reported in Table 1, unless otherwise specified): applying the QOF mortality benefit to all individuals aged 0–74 years as opposed to a concentrated (higher) mortality benefit to those with prevalent cardiovascular disease, high and low utility values, high and low cardiovascular disease prevalence and mortality multiplier estimates, and separate scenarios removing and doubling the annual average future health-care costs (Table 7 in the Appendix).

### Two-way sensitivity analysis

**Fig. 2:**
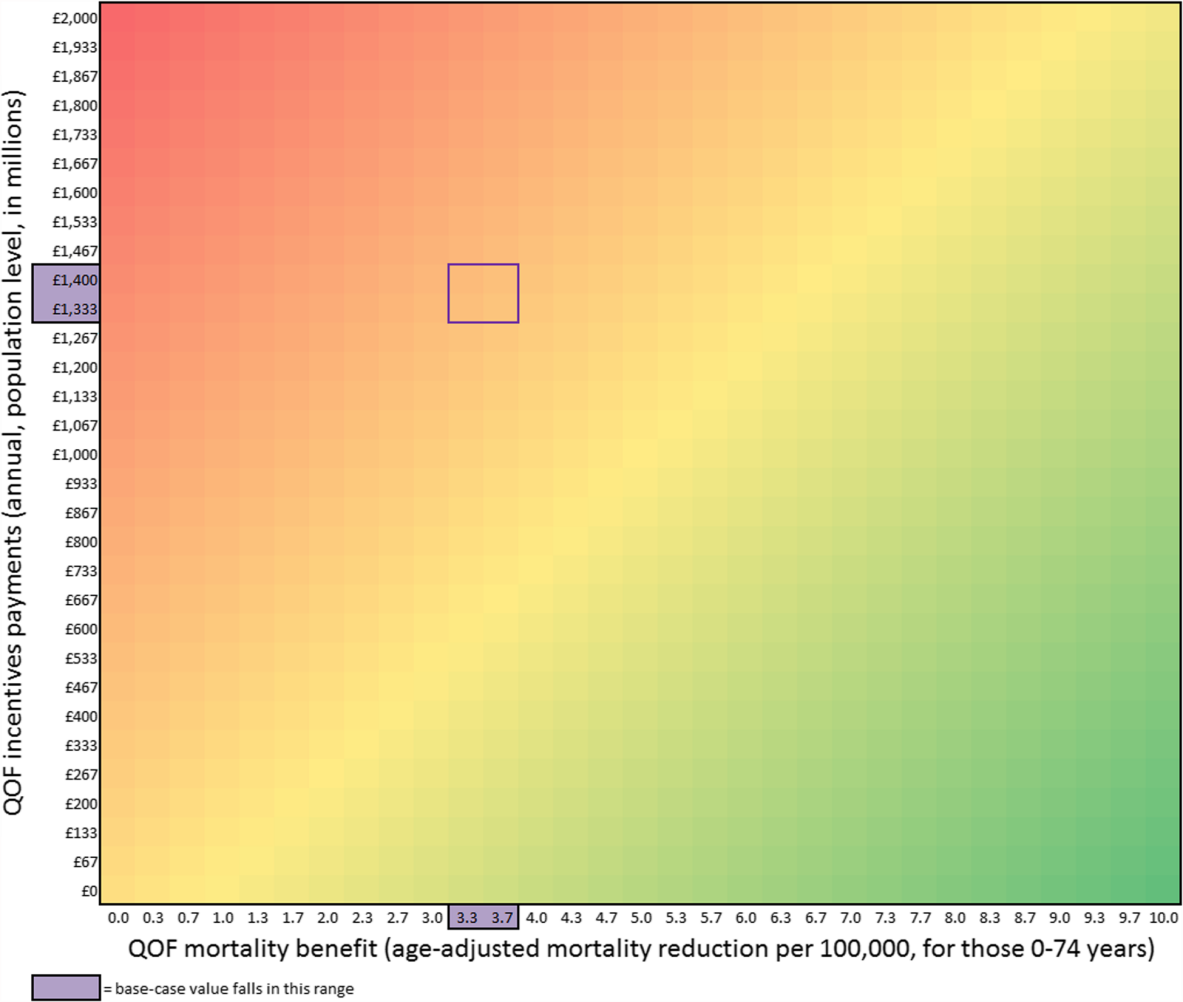
Two-way sensitivity analysis showing the optimal strategy for different combinations of the levels of QOF incentive payments and the QOF mortality benefit. The green regions show combinations of values that resulted in an ICER < £30,000/QALY for continuing the QOF compared to stopping the QOF, yellow indicates an ICER of £30,000/QALY and red indicates an ICER of >£30,000/QALY. ICER incremental cost-effectiveness ratio, QALY quality-adjusted life year, QOF Quality and Outcomes Framework

Fig. 2 shows the ICER as a function of the levels of QOF incentive payments and the QOF mortality benefit assuming a cost-effectiveness threshold of £30,000/QALY. The ICER was below thresholds of £30,000/QALY, £20,000/QALY and £13,000/QALY with QOF incentive payment levels of £893,979,617 (64% of the base-case value), £628,579,418 (45%) and £446,989,808 (32%), respectively. Similarly, the ICER was below thresholds of £30,000/QALY, £20,000/QALY and £13,000/QALY with QOF age-adjusted mortality reductions of 5.89 (160% of the base-case value), 8.25 (225%) and 11.59 (315%) respectively.

### Probabilistic sensitivity analyses

**Fig. 3:**
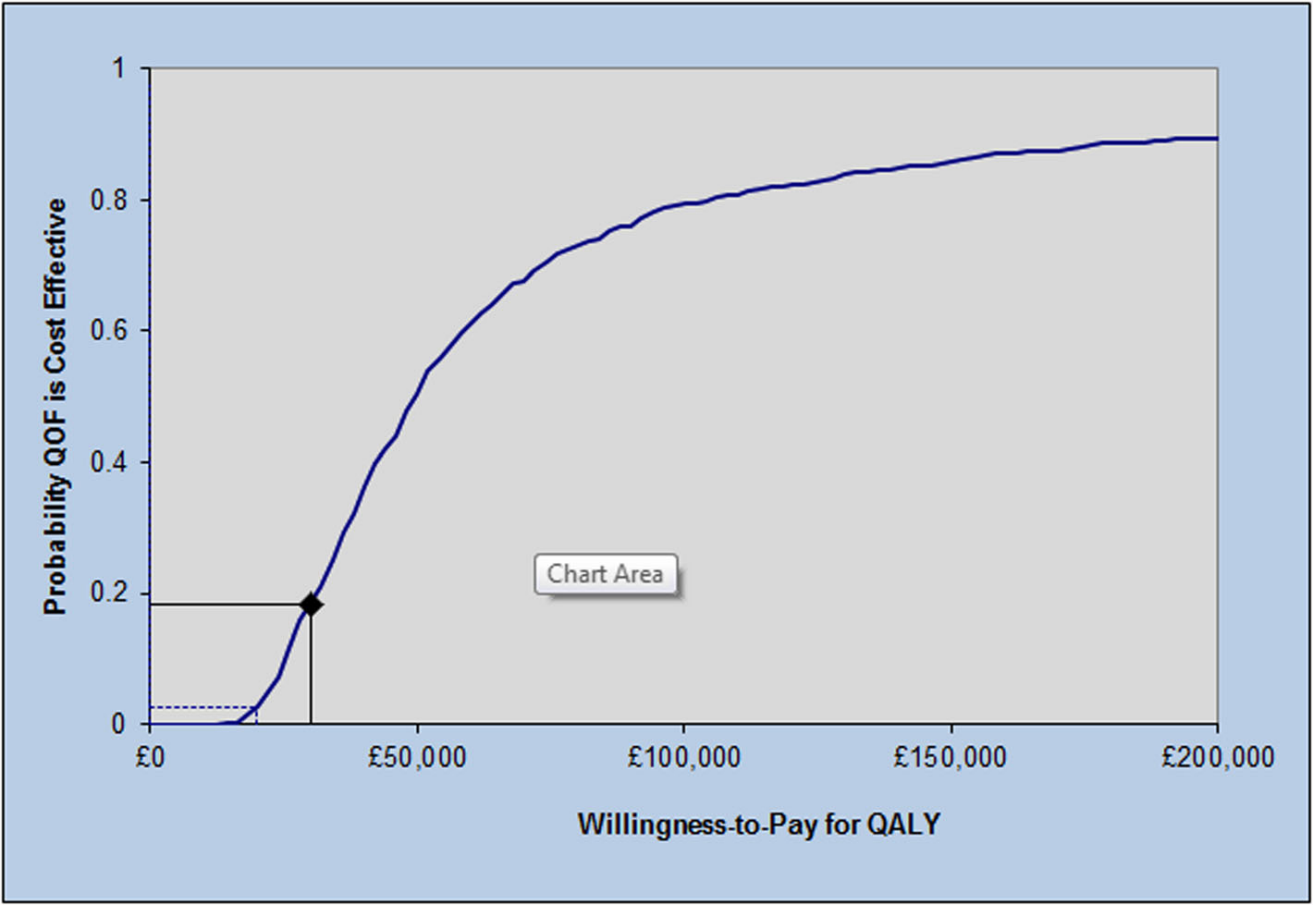
Cost-effectiveness acceptability curve for the probabilistic sensitivity analysis. The curve shows the probability that the QOF was cost-effective. It was calculated as the proportion of iterations with ICERs that were less than a given cost-effectiveness threshold. The health benefit (base-case value of 3.68 per 100,000 age-adjusted mortality reduction) was randomly drawn from a normal distribution (95% confidence interval −0.80 to 8.16). ICER incremental cost-effectiveness ratio, QALY quality-adjusted life year, QOF Quality and Outcomes Framework

**Fig. 4:**
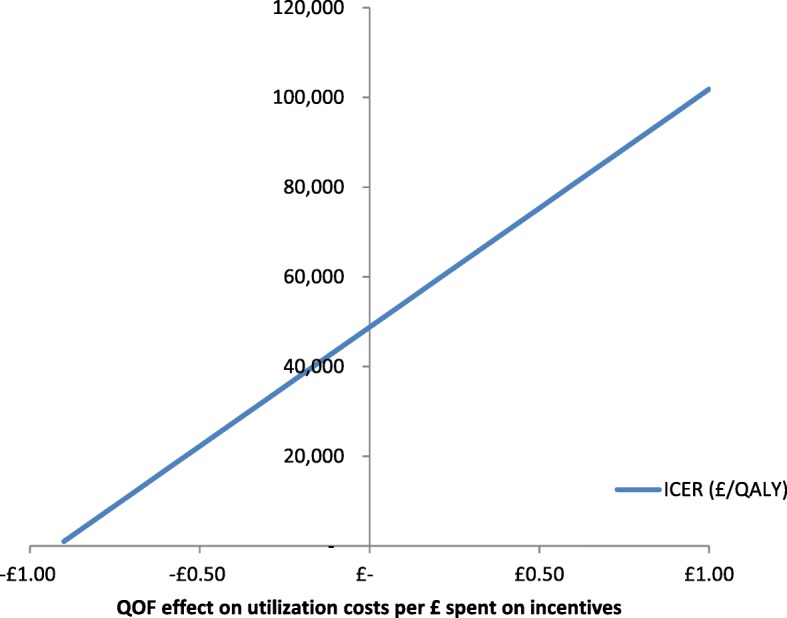
Cost-effectiveness ratio (£/QALY) for the QOF versus non-QOF as a function of QOF-incentivised utilisation costs. ICER incremental cost-effectiveness ratio, QALY quality-adjusted life year, QOF Quality and Outcomes Framework

**Fig. 5:**
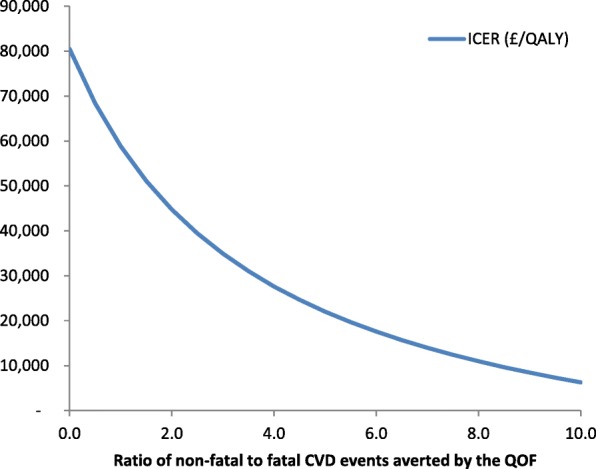
Cost-effectiveness ratio (£/QALY) for the QOF versus non-QOF as a function of non-fatal-to-fatal CVD events averted. CVD cardiovascular disease, ICER incremental cost-effectiveness ratio, QALY quality-adjusted life year, QOF Quality and Outcomes Framework

Fig. 3 shows the cost-effectiveness acceptability curve from the probabilistic sensitivity analysis. The QOF was cost-effective in 18.3%, 2.9% and 0.1% of probabilistic sensitivity analysis iterations using thresholds of £30,000/QALY, £20,000/QALY and £13,000/QALY, respectively. The 95% credible interval (i.e. the range where 95% of probabilistic sensitivity analysis results fell) for the ICER was £19,700/QALY to QOF dominated (which means continuing the QOF would result in higher costs and fewer QALYs compared to stopping the QOF).

## Discussion

In this study we modelled the cost-effectiveness of continuing the QOF in the UK to evaluate whether the incremental health gains from continuing the pay-for-performance programme would be worth the additional costs to do so. We found that the ICER for continuing the QOF was £49,362/QALY, with an 18% probability of being cost-effective in probabilistic sensitivity analysis using a cost-effectiveness threshold of £30,000/QALY. This estimate was robust to variation in assumptions related to non-fatal outcomes, increased drug costs and waning of benefits from the QOF. A probabilistic sensitivity analysis found that the QOF was cost-effective in only 18% of the scenarios tested. We found that ICERs of the QOF were substantially more favourable only in those scenarios where the QOF was associated with large reductions (beyond our base-case estimates) in (1) costs associated with averted health events or (2) non-fatal cardiovascular disease events. The estimated population opportunity cost of continuing the QOF (in terms of incremental net health benefit) was 226,109–979,917 QALYs lost.

Our estimate of the ICER for the QOF is above the conventional threshold of £20,000–30,000/QALY that is used to determine cost-effectiveness in the UK. This suggests that primary-care pay-for-performance in the United Kingdom has not been a cost-effective strategy to improve health. Nonetheless, our base-case analysis treats QOF incentive costs as incremental to the health system. We assumed that stopping the QOF would return all incentive payments to the NHS. However, if the NHS decided to stop the QOF and return all or some of the QOF payments to providers as increased capitation payments, as has already happened in Scotland, this would maintain the costs for the QOF while losing the benefits (unless the benefits from the QOF remain or wane over time after the financial incentives are stopped). Relative to this scenario, continuing the QOF would be more favourable. In sensitivity analyses, we found that continuing the QOF would be cost-effective (ICER < £30,000/QALY or < £13,000/QALY) if QOF incentive payments (see Fig. 2) were respectively 32% or 64% lower than our base-case estimate, assuming that the mortality impact of QOF remained unchanged. Our analysis gives policymakers cost-effectiveness information for all joint scenarios of QOF payment and mortality benefit estimates to facilitate decision-making around lower QOF payments considering potential reductions in QOF benefits that would follow from these decisions. Our results were not sensitive to the assumption of concentrating the QOF benefits in those with cardiovascular disease, as shown with an ICER > £30,000/QALY, even if assuming the QOF equally benefited all individuals aged 0–74 years with and without disease.

In general, cost-effectiveness analyses of pay-for-performance policies, especially those that use QALYs as the effectiveness measure (i.e. cost–utility analyses), are rare. In a systematic review of economic evaluations of pay-for-performance policies, Emmert and co-authors found only one cost–utility analysis [10]. That study, by Nahra and co-authors, modelled how hospital process improvements in heart-related care could be used to estimate QALYs via improved medication compliance and found that the pay-for-performance policy was cost- effective [13]. Since the publication of the review by Emmert et al., Meacock et al. and Walker et al. performed cost–utility analyses of pay-for-performance policies. Meacock and colleagues evaluated the cost-effectiveness of a pay-for-performance scheme for hospitals in the UK (the Advancing Quality programme) using reductions in 30-day mortality (among patients admitted for pneumonia, heart failure or acute myocardial infarction) estimated from a difference-in-difference study and found that the programme was cost-effective using a threshold of £20,000/QALY [11]. Walker and co-authors used previously published literature to estimate the potential cost-effectiveness of the QOF. They found that, for most QOF indicators studied, a less than 1% improvement would be needed for the programme to be cost-effective (using the £20,000–30,000/QALY threshold range). However, this study used estimates from randomised controlled trials to extrapolate the hypothetical effects of incentivising individual activities for which evidence on effectiveness was available, rather than estimating the impact of the overall programme [9].

Our study conflicts with previous attempts to estimate the cost-effectiveness of pay-for-performance policies. Only the Meacock et al. study used effectiveness estimates (reduction in mortality that was translated to QALYs gained) that were directly measured, as opposed to extrapolating intermediate outcomes (such as improvements in medication compliance) to QALYs. The Meacock cost-effectiveness study was based on a difference-in-difference analysis that found no significant impact of a hospital-based pay-for-performance programme on outcomes for two of the incentivised conditions (acute myocardial infarction and heart failure) and a modest improvement for the third (pneumonia) after 18 months [36]. However, this improvement was not sustained and re-analysis of the original data using a synthetic control approach found that the initial improvement for pneumonia was not statistically significant [37, 38]. Unlike the Meacock et al. study, we were not able directly to measure and thus include the administrative costs of running the pay-for-performance programme. Our study is the first to evaluate the cost-effectiveness of the QOF using direct estimates of mortality (as opposed to intermediate outcomes or hospital-based pay-for-performance policies).

Our study has several limitations, including four related to data limitations. First, we could not estimate administrative costs due to data limitations. If administrative cost data become available, these costs could be added to the annual incentive costs in our sensitivity analysis around costs (the horizontal axis in Fig. 2) to estimate the impact of these costs on the cost-effectiveness results. The addition of these administrative costs would increase the ICER for continuing the QOF. Second, although our model cohort was simulated until death, we restricted QOF mortality effects up to age 74 years given the source data [6]. Third, we also had limited information on the effect of the QOF on costs, such as those of additional visits to practices, referrals to secondary care and medication prescriptions. For instance, there are very few studies estimating the impact of the QOF on health-care utilisation, which is why we relied on a 2008 observational study from Scotland to estimate incentivised drug costs despite our model assuming a causal relationship between the QOF incentives and increased utilisation. To address this, we performed a sensitivity analysis around this input value, which showed that cost savings because of the QOF (i.e. averted utilisation costs outweighing the incentive costs) would be necessary to make the QOF cost-effective using a threshold of £30,000/QALY. Fourth, we also had incomplete information about the effects of the QOF on non-fatal outcomes, such as acute myocardial infarction and stroke. To address this, we varied our estimates across a range of assumptions about the effects of the QOF non-fatal outcomes and found that the ratio of fatal-to-non-fatal events would need to be more than doubled from our base-case estimate to make the QOF cost-effective. Fifth, our results from a probabilistic sensitivity analysis showed that there is considerable statistical uncertainty around the cost-effectiveness of continuing the QOF, suggesting that more precise estimates of the effect of the QOF on mortality could reduce the uncertainty around the decision of whether to continue the QOF. We also varied only one parameter (the effectiveness of the QOF) in the probabilistic sensitivity analysis because it was the only input with a well-estimated 95% confidence interval. Adding other parameters to the probabilistic sensitivity analysis could produce a higher uncertainty in our cost-effectiveness results, but that would not change the overall conclusion of our analysis. Sixth, QOF is subject to annual review and amendment [6–8, 24, 26], and our main estimates for the effectiveness and incentives costs were based on the first 7 years of the programme. Therefore, our analysis is evaluating the decision to continue with incentives contained in QOF from 2004 to 2010 versus discontinuing these incentives. We have not evaluated the most recent versions of the QOF, for which we do not have linked cost and effectiveness data, but incentives for the conditions of interest were retained in these versions.

Despite these limitations, our findings imply that the UK should redesign the QOF or pursue alternative interventions to improve population health efficiently. The QOF is already in transition. The programme was reduced in scope in 2014, with 40 indicators retired to focus on a set of 83 key indicators [7, 39]. In Scotland, the QOF was withdrawn altogether in 2016, with practices continuing to receive payments based on their historical performance without any further need to meet QOF targets. In the future, quality improvement in Scottish practices will be managed by local peer support networks and will rely on clinical governance arrangements rather than financial incentives [40]. NHS England is also now seeking to develop a successor to the QOF [41]. To enable informed decisions on further redesign or replacement of the QOF, future research should compare the cost-effectiveness of the programme with alternative system-level interventions (whether these programmes are different forms of financial incentives for providers or patients, or use other mechanisms to improve quality, costs or access to care). Similar research should be undertaken in other settings where pay-for-performance has been implemented, comparing its cost-effectiveness with other health system-level policies such as value-based insurance design [42], computerised decision support interventions [43] or value-based outcome reporting tools [44]. These future analyses would provide crucial information about whether pay-for-performance in primary care is a cost-effective way to improve population health.

## Conclusions

Compared to stopping the QOF and returning all associated incentive payments to the NHS, continuing the QOF is not cost-effective. To improve population health efficiently, the UK should redesign the QOF or pursue alternative interventions.

### Additional file

Additional file 1: The model, which is the full simulation model used to perform the cost-effectiveness analyses, is programed in Microsoft Excel with Visual Basic for Applications (VBA). (XLSM 648 kb)

## Appendix

**Table 4:** Estimates of incentive payments to UK practices under the Quality and Outcomes Framework (inflated to 2016£)

Year	England^a^	Northern Ireland^a^	Scotland^b^	Wales^a^	Total
2004/5	£830,220,692	£36,069,236	£90,923,805	£48,008,831	£1,005,222,565
2005/6	£1,350,199,371	£58,115,719	£155,248,597	£79,707,784	£1,643,271,471
2006/7	£1,237,234,310	£53,602,369	£144,140,152	£73,024,636	£1,508,001,467
2007/8	£1,213,821,483	£52,481,420	£142,189,299	£72,081,589	£1,480,573,791
2008/9	£1,147,372,625	£49,335,331	£135,246,153	£67,898,096	£1,399,852,206
2009/10	£1,128,185,395	£48,508,555	£132,825,069	£66,939,222	£1,376,458,241
2010/11	£1,122,907,420	£46,715,424	£128,483,891	£66,415,581	£1,364,522,315
Total	£8,029,941,295	£344,828,055	£929,056,966	£474,075,739	£9,777,902,056

^a^Based on estimates from the Information Centre for Health and Social Care [24, 26]

^b^Based on payment data from the Information Services Division Scotland [27]

**Table 5:** Drug classes, changes in utilisation and annual prices

Drug class^a^	DDD/PU/mo^b^	Annual price^c^
Lipid regulating drugs	1.92	61.57
Renin angiotensin	0.84	37.29
Thiazides/diuretics	0.24	5.66
Oral antidiabetic	0.14	42.79
Antiplatelet	0.33	14.77

*DDD* defined daily dose, *mo* month, *PU* prescribing unit, *QOF* Quality and Outcomes Framework

^a^Cardiovascular disease drug classes from MacBride-Stewart et al. [29]

^b^Increase in defined daily doses (DDDs) per prescribing unit (PU) per month of QOF-related drugs

^c^Weighted average price (weights based on quantity dispensed by specific drugs and doses)

(source: Health and Social Care Information Centre. Prescription cost analysis, England 2012)

https://digital.nhs.uk/data-and-information/publications/statistical/prescription-cost-analysis/prescription-cost-analysis-england-2012

**Table 6:** Population-level incremental net health benefit results

			Net health benefit using different cost-effectiveness thresholds (QALYs)		
	Discounted QALYs	Discounted costs	£30,000/QALY threshold	£20,000/QALY threshold	£13,000/QALY threshold
Stopping the QOF	39,966,375	£0	39,966,375	39,966,375	39,966,375
Continuing the QOF	40,316,707	£17,293,239,670	39,740,266	39,452,046	38,986,458
Delta	350,332	£17,293,239,670	−226,109	−514,330	−979,917

*QOF* Quality and Outcomes Framework, *QALY* quality-adjusted life year

**Table 7:** One-way sensitivity analysis results

Variable	Base-case value	Sensitivity analysis range	ICER at low value	ICER at high value
Adjusted QOF mortality benefit (for those aged > 40 years with CVD)	58.93	−130.57 to 12.81	QOF dominated	£20,044/QALY
CVD prevalence, males (aged 45–64 years, aged 65–74 years)	14.6%, 28.5%	±20%	£49,266/QALY	£49,485/QALY
CVD prevalence, females (aged 45–64 years, aged 65–74 years)	8.4%, 22.5%	±20%	£49,302/QALY	£49,414/QALY
CVD mortality multiplier (male, female)	1.6, 2.1	±20%	£47,246/QALY	£51,249/QALY
CVD utility	0.796	±20%	£43,516/QALY	£64,805/QALY
Acute CVD event costs (i.e. costs within first year of CVD event)	£10,871	±20%	£49,850/QALY	£48,874/QALY
Chronic CVD event costs (i.e. annual costs for all years after first year)	£3282	±20%	£50,350/QALY	£48,375/QALY
Average NHS costs by age	Age-based table	£0 to +100%	£46,270/QALY	£52,455/QALY
Discount rate	3.5%	0–5%	£38,337/QALY	£54,587/QALY

Base-case ICER of £49,362/QALY; base-case values for each variable are reported in Table 1 in the main text

*CVD* cardiovascular disease, *ICER* incremental cost-effectiveness ratio, *NHS* National Health Service, *QALY* quality-adjusted life year, *QOF* Quality and Outcomes Framework

## Abbreviations

BHF: British heart Foundation
CEAC: Cost-effectiveness acceptability curve
DDD: Defined daily dose
EQ-5D: EuroQOL 5 dimensions
ICD: International classification of diseases
ICER: Incremental cost-effectiveness ratio
NICE: National Institute for health and care excellence
NHS: National health service
PU: Prescribing unit
QALY: Quality-adjusted life year
QOF: Quality and Outcomes Framework
UK: United Kingdom
US: United States
